# Calprotectin as new potential clinical marker for multiple myeloma

**DOI:** 10.1371/journal.pone.0282841

**Published:** 2023-03-16

**Authors:** Parisa Khosravi, Saeid Abroun, Saeid Kaviani, Saman Masoudifar, Homayoun Sarough Farahani

**Affiliations:** 1 Department of Hematology, Faculty of Medical Sciences, Tarbiat Modares University, Tehran, Iran; 2 Arak university of Medical Science, Arak, Iran; Bari University Aldo Moro, ITALY

## Abstract

Increased levels of inflammatory cytokines in multiple myeloma (MM) patients and the role of inflammation in disease pathogenesis, have recently been considered. The aim of this study was to quantitatively evaluation of fecal calprotectin (CP) as a non-invasive biomarker for the evaluation of inflammation in patients with multiple myeloma. This study is a hospital-based case control study. MM patients referred to patients referred to medical centers of Tehran province, Iran, were identified and classified into two groups of new MM patients (n = 40) and patients undergoing treatment (n = 28). Healthy individuals were included in the study as healthy control (n = 25). Morning stool samples were collected and CP was extracted immediately. After collecting the samples, CP was measured according to ELISA method and was determined in μg/g of feces. Values ​​above 50 μg/g of feces are positive and indicate inflammation. The results revealed that there is a significant difference between groups in terms if CP mean (p = 0.001). The mean of CP among new cases, under treatment and control groups were 301.3 (SD: 141.0), 165.1 (SD: 153.9) and 36.9 (SD: 13.5), respectively. Then the groups were compared in pairs, the results showed that the new case group was significantly different from the under-treatment group (p = 0.001), and also the control group showed a significant difference with the new case group (p = 0.001) and the under-treatment group (p = 0.001) that the amount of CP in the control group was significantly lower than the other two groups. In addition, the results of the study showed a significant correlation between age and plasma cells with CP value, so that with increasing age and plasma cells, CP value also showed a significant increase. The results indicate that quantitative evaluation of CP as a non-invasive laboratory biomarker has a high potential as a clinical marker in patients with multiple myeloma and inflammation should considered as a hallmark of cancer. Further diagnostic studies are recommended.

## Introduction

Multiple myeloma (MM) is a bone marrow malignancy, which characterized by the presence of abnormal proliferation of plasma cells in the bone marrow with potential for uncontrolled growth, can lead to destructive bone lesions, anemia, renal failure, hypercalcemia, etc. MM is diagnosed in about 588,161 people worldwide each year [1–3]. Diagnosis of MM requires the presence of clinical complications and organ damage due to clonal abnormalities and neoplasm proliferation of plasma cells such as hypercalcemia, renal failure, anemia, and bone lesions [4].

The supporting structure of the bone marrow plays a key role in the development of malignancies by interacting and inducing signaling pathways in stem cells. In fact, the interaction of cell and peripheral matrix of bone marrow (including cytokines, interleukins, and growth factors) is involved in the development of hematologic malignancies. The most important cellular interactions with bone marrow space that lead to the activation of important signaling pathways such as nuclear factor kappa B (NFkB) are: interleukin-6 (IL6), insulin-like growth factor-1 (IGF-1), Stromal cell-derived factor-1 (SDF-1), tumor necrosis factor-alpha (TNF-α), interleukin-8 (IL8), interleukin-17 (IL17), and vascular endothelial growth factor (VEGF), which are known to play a role in inflammation, immunosuppression, tumorigenesis, and osteolysis [5]. Among these cytokines, interleukin 6 plays a key role in the survival and proliferation of malignant myeloma cells. The two main sources of autocrine and paracrine secretion of interleukin-6 are malignant myeloma cells themselves and bone marrow niche stromal cells, respectively. Interleukin 6 is one of the most important inflammatory cytokines. In general, inflammation in patients with multiple myeloma has an important role in the progression and pathogenesis of the disease [6].

Calprotectin (CP), a 36 kDa heterodimer protein (consisting of 2 proteins S100A8 and S100A9) is a calcium- and zinc-binding protein with low molecular weight [7, 8]. It seems that the antimicrobial properties of CP are due to the ability of this protein to chelate the metal ions required by pathogens. In addition, CP, as a damage-associated molecular patterns (DAMPs) molecule by binding to toll-like receptor 4 (TLR4) on immune cells, is involved in initiating the immune and inflammatory response [9–11].

CP is known as the acute phase protein because it is increased in many inflammatory diseases including inflammatory bowel disease (IBD), irritable bowel syndrome (IBS), bacterial infections and transplant rejection. The gene locus of this protein on the human chromosome is 1q21. CP is found in many cells of the body, including neutrophils, monocytes, keratinocytes, and macrophages. Neutrophils, however, are known to be the main cell producing and source of CP [12, 13].

The main receptors for CP in the body are RAGE (the receptor for advanced glycation endproducts), TLR4 and extracellular matrix metalloproteinase inducer (EMMPRIN). All of which induce NFkB and MAP signaling pathways and lead to proliferation, survival, and inflammation [14–16]. Increased CP has been shown to be directly related to increased ESR and CRP [17].

Previous studies have shown that an increase in CP is significantly associated with cancer stage. CP has been shown to facilitate tumor cell implantation by increasing the expression and secretion of growth factors, such as TGF and VEGF. On the other hand, studies show that CP has an anti-tumor role by activating caspases 3, 7 and 8 in target cells [18–20].

Since CP is considered as an inflammatory marker, and on the other hand, inflammation is considered as one of the main causes of MM progression, our hypothesis in this study was that there is a relationship between fecal CP level and the development and progression of MM. Based on our search, no similar study has been designed so far regarding the relationship between CP level and MM, and also the assessment of fecal CP in patients is considered as an inexpensive and non-invasive test, which is more compliant and accompanied by patients.

To the best of our knowledge, no study has been performed to investigate intestinal inflammation in patients with MM and due to the pathophysiology of MM, this study aimed to quantitatively evaluate fecal CP in patients with MM at different stages of the disease and compare it with the control group.

## Materials and methods

### Study design

This study is a hospital-based case control study in which three groups were included. Two groups of patients including newly diagnosed (new case group, (n = 40)) and under treatment patients (n = 28) and a group of healthy individuals (n = 25) who were age-matched to the two groups of patients were included in the study.

### Ethical consideration

This study was approved by Ethical Committee of Tarbiat Modares University on January 01, 2021 with ethical code of: IR.MODARES.REC.1399.194. However, we obtained a written informed consent to participate in the study.

### Participants

The newly diagnosed group included patients referred to medical centers of Tehran province, Iran, who were diagnosed with multiple myeloma. The definitive diagnosis of the disease is in accordance with International Myeloma Working Group (IMWG) criteria and approved by the pathologist of the center. The under-treatment group were patients with multiple myeloma undergoing chemotherapy and hospitalized in medical centers of Tehran province, Iran, in 2021, who were diagnosed as same as the first group. The control group included people who did not have multiple myeloma and did not report other related underlying diseases.

### Inclusion and exclusion criteria

Inclusion criteria included having written informed consent to participate in the study, age over 5 years, no gastrointestinal infections and gastrointestinal cancers, no use of nonsteroidal anti-inflammatory drugs (NSAIDs) and proton pump inhibitors, no untreated food allergies and those has not developed gastrointestinal inflammation such as inflammatory bowel disease (IBD), autoimmune enteropathy, celiac disease (untreated), ulcers, gastrointestinal bleeding over 100 ml per day, gastroesophageal reflux disease, cirrhosis, cystic fibrosis and adolescent polyps. Patients who did not want to continue the study were excluded from the study.

### Stool sample collection and storage of samples

The most common target sample for CP levels is a stool sample. The concentration of CP in stool is 6 times that of other human biological samples, and its increase in fecal samples during inflammatory diseases is proportional to its increase in other biological fluids in the body. Some studies have shown circadian changes in fecal CP, possibly due to changes in bowel movements. Therefore, a morning sample is recommended for data uniformity [21]. The preparation of a random stool sample (preferably the first morning sample) was collected in sterile stool containers without the addition of additional material [22]. In this study, fecal samples were analyzed immediately after delivery to the laboratory and the process of extracting CP was performed on them. After centrifugation, the supernatant containing CP is placed in a -20 freezer, and all samples were analyzed by ELISA before the end of 6 months.

### Step-by-step extraction process of fecal CP

Prior to extraction, the sample was well homogenized using a wooden spatula.

1. After reaching the extraction buffer temperature inside the kit and samples at room temperature, 2 liters of buffer was added to each tube.
2. The spiral loop of the sample located in the tube cap was dipped five times in different places of the fecal sample to a depth of 5 mm and about 40 mg of the sample was collected.
3. The loop was taken into a tube containing the extraction buffer and after 20 minutes of strong shaking or vortex the sample was completely dissolved in the extraction buffer.
4. The tubes were centrifuged at 3000xg for 3 minutes and the supernatant was poured as a sample taken into another test tube and the precipitate was discarded as infectious waste.
5. The extracted sample was stored at a temperature of 2 to 8° C for a maximum of 5 days and at a temperature of -20° C or lower for a long time.

### Step-by-step process of CP ELISA

Calprotectin ELISA test was performed according to the instructions of Pishtazteb Kit (Iran, Tehran, Pishtazteb co.). The ELISA test designed in this kit is a sandwich ELISA.

1. Stool samples were diluted 1: 50 using a sample dilution buffer.
2. 100 μl of each standard, control sample and diluted samples were poured into each well (standards and samples were repeated).
3. Cover the wells with a special plate label and place the microplate on a shaker for 30 minutes at a speed of about 500 rpm at room temperature (22 to 28 centigrade).
4. Each well was washed 5 times with 300 microliters of solution.
5. 100 μl of Enzyme Conjugated Calprotectin-Anti solution was added to each well.
6. Cover the wells with a special plate label and place the microplate on the shaker for 30 minutes at a speed of about 500 rpm at room temperature (22 to 28 degrees Celsius).
7. Each well was washed again 5 times with washing solution.
8. 100 microliters of dye solution (Substrate—Chromogen) was added to each well.
9. The wells were placed for 15 minutes at room temperature and in the dark.
10. By adding 100 μl of solution stop to each well, enzymatic reactions were stopped.
11. To measure the light absorption, an electrifier with a 450 nm filter was used.

### Statistical analysis

Mean (standard deviation) and count (percentage) were used to describe quantitative and qualitative data, respectively. The normality assumption of the data was checked using Shapiro-Wilk test and based on the obtained results, an appropriate parametric or nan-parametric test was chosen to test the hypothesis. The qualitative data were analyzed by likelihood ratio chi square test. To test the qualitative data in case of normal distribution of data, one way analysis of variance (ANOVA) and paired sample t-test was used. In cases where the data distribution was not normal, two-sample Wilcoxon rank-sum (Mann-Whitney U) test and Kruskal-Wallis equality-of-populations rank test was used. In addition, to assess the linear relationship between quantitative data, the Pearson correlation coefficient was used. All analyzes were performed using Stata software version 16 (Stata Corp, College Station, TX, USA) and at a significance level of 0.05.

## Results

In this case control study, 93 cases were included in the analysis. Three groups were compared in terms of age and gender distribution and the related results were presented in Table 1. The results suggested that the three groups have a similar gender distribution (p = 0.606) and also there are no significant difference between the mean age of study groups (p = 0.152).

**Table 1:** Comparison of mean age and gender distribution among three study groups.

Variables		New cases (n = 40)	Under treatment (n = 28)	Control (n = 25)	P
Age	Mean (SD)	67.0 (8.2)	62.3 (10.8)	64.7 (10.7)	0.152
Gender	Female	14 (35%)	13 (46.4%)	9 (36.0%)	0.606
	Male	26 (65%)	15 (53.6%)	16 (64.0%)	

**Fig 1:**
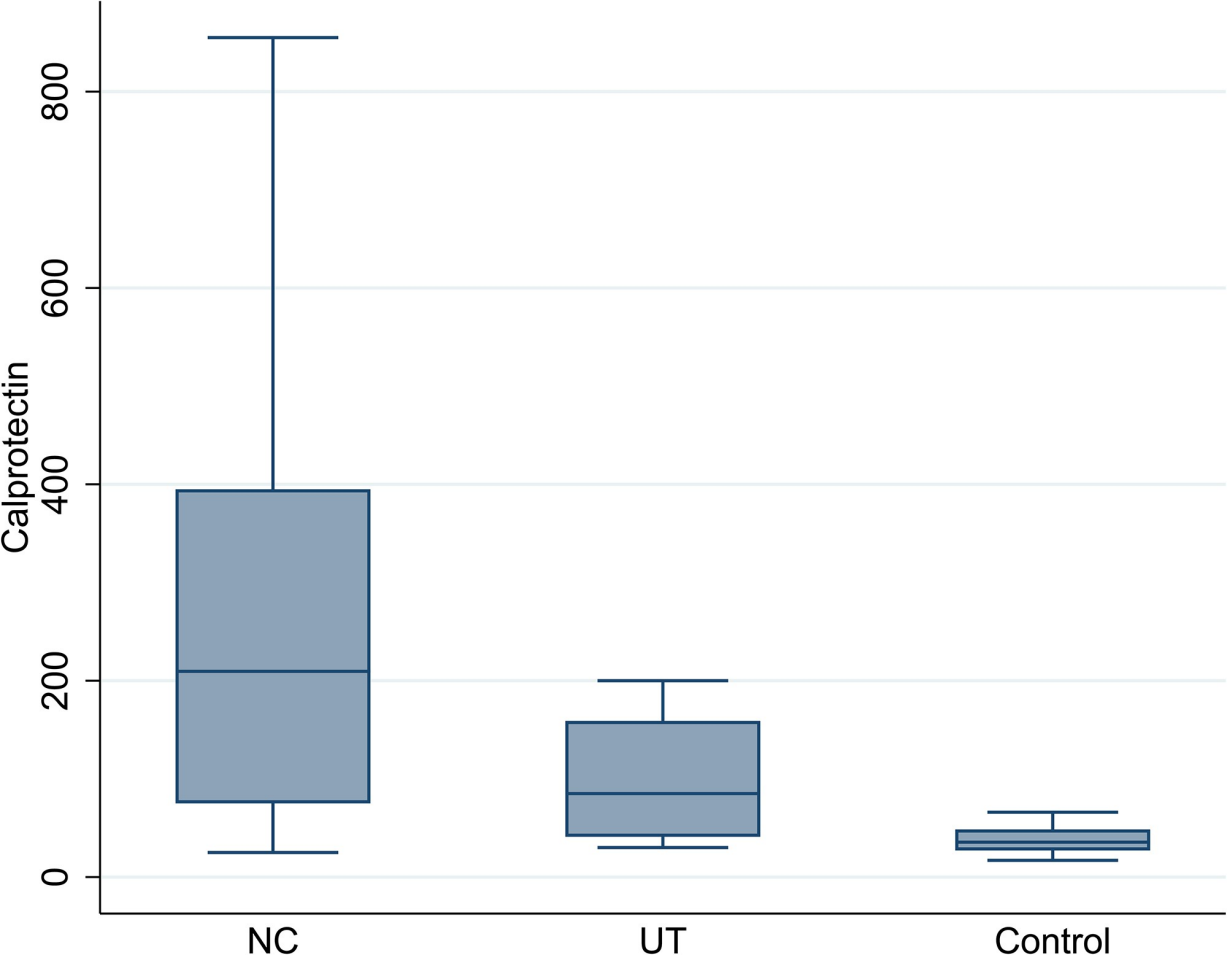
The comparison of mean of CP among three study groups.

The mean of CP among three groups was compared and displayed in Fig 1. As it was showed in Fig 1, the results revealed that there is a significant difference between groups in terms if CP mean (p = 0.001). The mean of CP among new cases, under treatment and control groups were 301.3 (SD: 141.0), 165.1 (SD: 153.9) and 36.9 (SD: 13.5), respectively. Then the groups were compared in pairs, the results showed that the new case group was significantly different from the under-treatment group (p = 0.001), and the control group showed a significant difference with the new case group (p = 0.001) and the under-treatment group (p = 0.001) that the amount of CP in the control group was significantly lower than the other two groups. In the new case group, 100% (n = 40) of the participants and in the under-treatment group 78.5% (n = 22) had a CP value higher than 50 μg/g, while this value was 20% (n = 5) in the control group and in this regard, a significant difference was observed between the three groups (p = 0.001).

**Fig 2:**
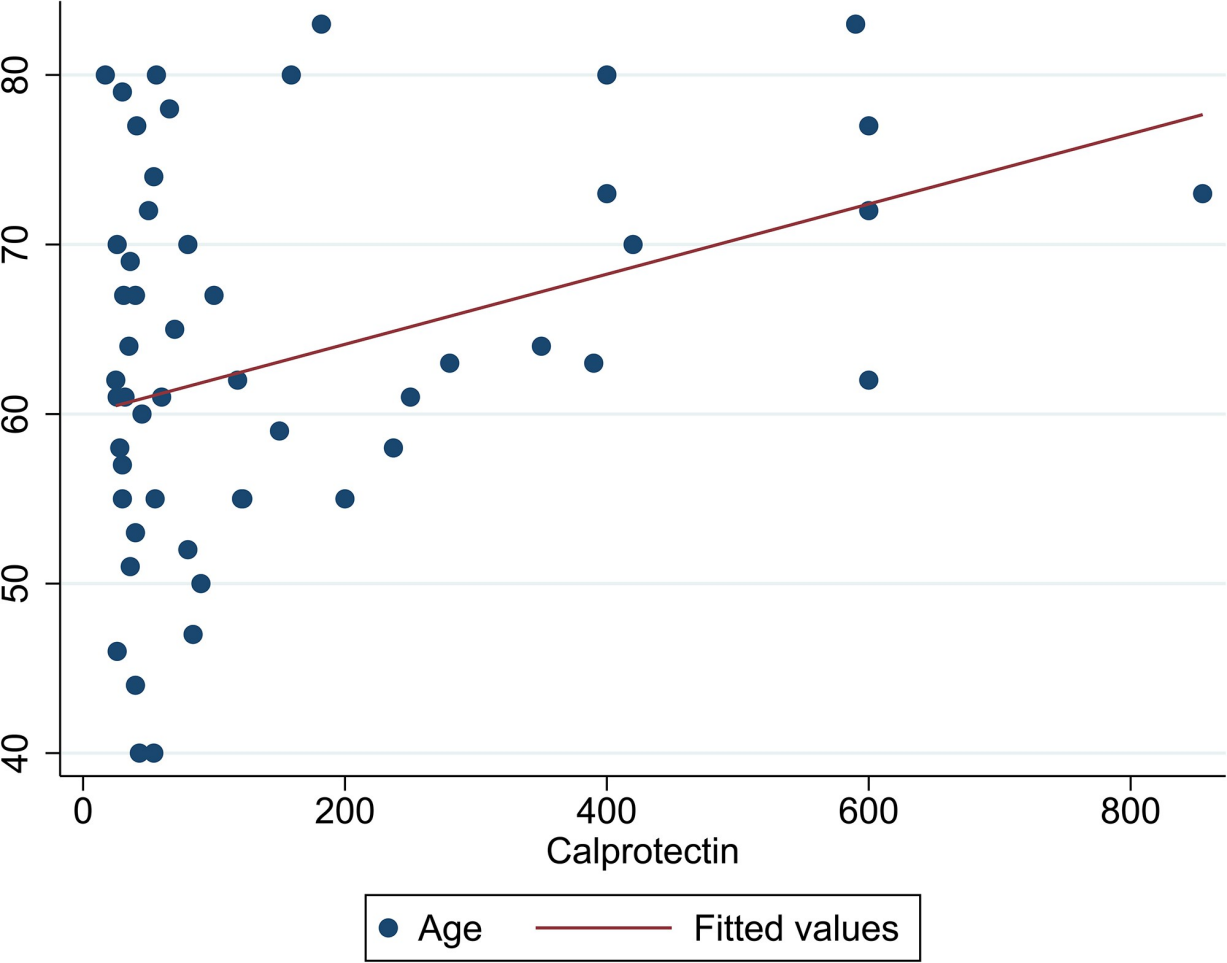
The correlation of age and CP among patients with MM (n = 68).

**Fig 3:**
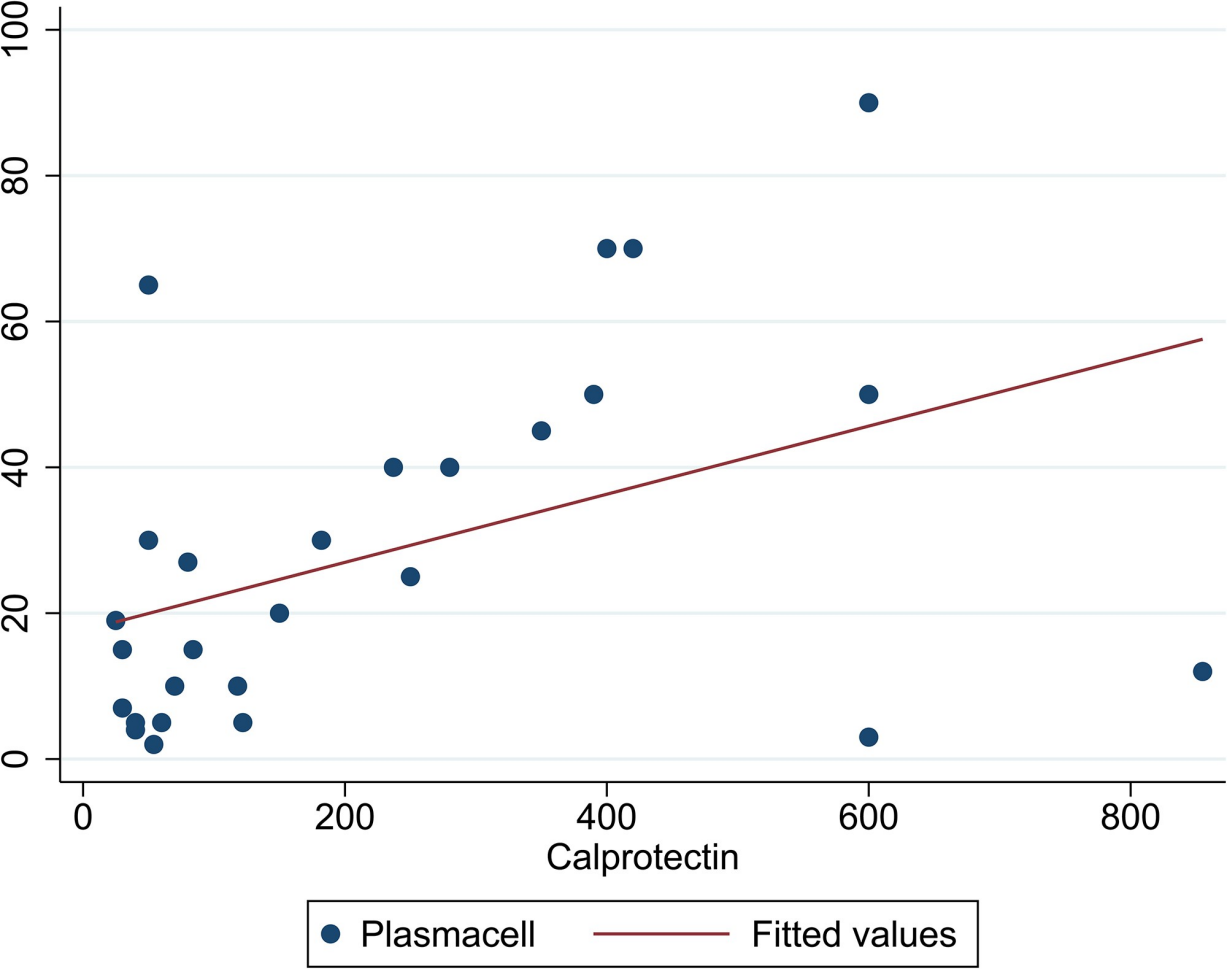
The correlation of plasma cell and CP among patients with MM (n = 68).

As shown in Fig 2, among patients with MM, the correlation between age and CP was examined and the results showed a positive and significant correlation between age and CP (correlation coefficient = 0.375, p = 0.001). In addition, the correlation between plasma cell and CP amount was examined in patients with MM (Fig 3), which showed a positive and significant correlation between plasma cell and CP amount (correlation coefficient = 0.656, p = 0.001).

However; we reassessed the CP level among 10 new cases after treatment and the results suggested that the mean of CP after treatment (mean: 315.4, S.D: 116.9) increased significantly (p = 0.001) compared to before treatment (mean: 182.0, S.D: 87.5) in these cases.

## Discussion

The most important findings of this study supported the hypothesis that CP levels are increased in patients with MM and the results suggested the higher CP level in new case group followed by under treatment and control group and the mean of CP in control group was significantly lower than both new case and under treatment group. In addition, the results of the study showed a significant correlation between age and plasma cells with CP value, so that with increasing age and plasma cells, CP value also showed a significant increase. In addition to the above, the analysis of CP level among 10 new cases revealed that the mean of CP increased significantly after treatment compared to before treatment.

The decrease in calprotectin concentration in a group of new case patients, after receiving treatment, has been associated with the entry of these patients into the remission stage. On the other hand, a noticeable increase in calprotectin in one patient under treatment (after 24 months of treatment) was associated with symptoms of disease recurrence (increase in graded dysplasias, anemia, and peak gamma). Therefore, it is possible that calprotectin changes during the treatment of multiple myeloma patients are directly related to the response to treatment, the severity of the disease and even the prediction of disease recurrence, which definitely needs more studies in this field.

In addition, the examination of diagnostic data of patients shows a direct relationship between stool calprotectin concentration and other clinical biomarkers such as acute phase proteins, ESR and gamma globulin (in serum electrophoresis). However, due to the limitation in the number of samples and the incomplete clinical data of the patients, it was not possible to conduct a detailed investigation and statistical analysis in this regard.

Until recent years, most studies have been focused on fecal CP as a laboratory biomarker with high sensitivity and specificity in inflammatory diseases of the gastrointestinal tract [23–25] but recently, evaluation of this biomarker and its possible role in the pathogenesis of other diseases and malignancies have been discussed [26–32]. To the best of our knowledge, no similar study has been performed on fecal calprotectin level in patients with MM, so this study was designed to revealed the possible association.

This study showed that fecal calprotectin level was significantly higher in the new case group than the treated group and the control group, and this value was significantly higher in the treated group than the control group. Also, the fecal calprotectin level in the new case group was higher than the treated group. These results emphasize that fecal calprotectin level increases after the onset of MM and after treatment, its level can be reduced.

In line with our study, elevated calprotectin has been observed in neonatal infections, early stages of lung infection, early stages of heart and kidney transplant rejection, and in acute and chronic GVHD (graft versus host disease) in bone marrow transplant patients [33, 34]. In 2017, Muhammet Fatih Topuz et al. showed that serum calprotectin in patients with laryngeal cancer was significantly higher than in patients with benign laryngeal tumors, and evaluation of this protein as a diagnostic and prognostic biomarker was recommended [18]. In 2019, Krečak et al. [35] with demonstrating an increase in serum calprotectin in Philadelphia chromosome negative myeloproliferative patients, hypothesized that increased Jak stat signaling pathway activity in neutrophil and its constant stimulation to the secretion of inflammatory proteins, has led to an increase in serum calprotectin. This study was the first attempt to evaluate calprotectin as a laboratory biomarker in blood malignancies.

Elevated calprotectin levels have been shown in inflammation, neoplastic tumor cells and various human cancers. Many cancers are caused by infection, chronic irritation, and inflammation, as studies show. The inflammatory microenvironment, which mainly contains calprotectin, plays an important role in increasing inflammatory factors and the neoplastic process and metastasis [12]. In the present study, quantitative evaluation of fecal calprotectin was performed for the first time in patients with MM. An increase in calprotectin was observed in 100% of new cases and 78.5% of under treatment cases at the time of diagnosis (before treatment). The mean of fecal Calprotectin among new cases is 8.1 times that of calprotectin in the control group (301.3 vs 36.9), which indicates a widespread increase in inflammation in these patients.

One of the most important cells in supporting myeloma cells is neutrophils. NET (neutrophil extra traps) increases TLR (toll-like receptor) expression by inducing NFkB pathways in plasma cells [36]. Increased expression of TLR on malignant myeloma cells was shown in previous studies [37]. The most important TLRs expressed in malignant plasma cells are TLR 4, TLR 9, TLR 8, TLR7 and TLR 10. Induction of myeloma cell proliferation, drug resistance to dexamethasone with TLR agonists and correlation of TLR expression with IL6 secretion indicates the important role of this inflammatory receptor in the pathogenesis of MM [38].

In this study, the relationship between the increase of CP and the increase of bone marrow plasma cells in MM patients has been shown. There is a possibility of the role of CP as a factor in the occurrence of the disease and also as a clinical marker in the progression of the disease.

In 2021, Ribatti et al. [39], showed that the best strategy for cancer treatment is based on the increased hallmarks of cancer (tumor) in each person, such as factors related to tumor cell proliferation, angiogenesis factor, chronic inflammation, and immune tolerance. The current study shows the value of identifying tumor hallmarks. Based on this study, by examining the increase of CP in MM patients, therapeutic strategies can be used to control inflammation, and by monitoring this hallmark, the inflammation and subsequent progression of MM can be monitored in this patients.

In addition, new therapeutic approaches based on epigenetic targets such as miRs and their carriers (liposomes, polymerase and exosomes) in phase I/II clinical trials have shown promising results in preventing the progression of MM and inhibiting drug resistance. Investigating CP at the epigenetic level and investigating the effective miRs on its expression can identify this marker as an effective inflammatory factor in the development and occurrence of the disease for therapeutic purposes as an innovative therapeutic approach in nano-medicine [40].

Due to the possible role of calprotectin in supporting myeloma of bone marrow, in this study, based on the results of pathology and flow cytometry of patients, the percentage of bone marrow plasma cells was calculated. Statistical analysis showed a significant correlation between calprotectin levels and increased bone marrow plasma cells in these patients.

Patients in the treatment group were in different periods of the treatment process. The duration of treatment in these patients varied from 5 to 120 months. In this group, calprotectin increased compared to the control group. It seems that the decrease in calprotectin in this group of patients is related to the response to treatment and the stage of the disease. One of the major known receptors for calprotectin in the immune system is TLR4 [12]. As mentioned earlier, studies show an increase in TLRs in myeloma cells. Based on this, it can be said that a sharp increase in calprotectin in some patients undergoing treatment can lead to disease recurrence, and quantitative evaluation of this non-invasive biomarker has prognostic value in these patients.

The strength of this study is that in this study, for the first time, the relationship between CP and MM has been investigated. One of the limitations of this study is that this study was performed in a single center and on a limited number of patients, so the generalization of the results should be done with caution. It is suggested that by carefully studying the role of calprotectin in the pathogenesis of MM in in-vitro, in addition to the diagnostic role of this protein, it can be used as a therapeutic target in these patients. Another limitation of this study was that the role of CP was not investigated at the genomic level and the study of genetic, epigenetic pathways, micro RNAs and miR carriers (liposome, polymerase, and exosome) are recommended in the further studies. It is also recommended to carefully examine the relationship between fecal calprotectin levels and other prognostic criteria such as albumin, beta 2 microglobulin, and known cytogenetic characteristics in patients with MM.

## Conclusion

The results indicate that quantitative evaluation of CP as a non-invasive laboratory biomarker has a high potential as a clinical marker in patients with multiple myeloma and inflammation should considered as a hallmark of cancer. Further diagnostic studies are recommended.

## Supporting information

S1 Data The used data in the paper. (XLSX)
